# Author Correction: Social attraction in *Drosophila* is regulated by the mushroom body and serotonergic system

**DOI:** 10.1038/s41467-020-19719-4

**Published:** 2020-11-06

**Authors:** Yuanjie Sun, Rong Qiu, Xiaonan Li, Yaxin Cheng, Shan Gao, Fanchen Kong, Li Liu, Yan Zhu

**Affiliations:** 1 State Key Laboratory of Brain and Cognitive Science, Institute of Biophysics, Chinese Academy of Sciences, 15 Datun Road, Beijing, 100101 China; 2 University of Chinese Academy of Sciences, Beijing, 100049 China; 3 Advanced Innovation Center for Human Brain Protection, Capital Medical University, Beijing, China; 4 Present Address: New England Biolabs (Beijing) LTD., No.1 Wang Zhuang Road, Beijing, China; 5 Present Address: Shenzhen Science Museum, Shangbu Road, 1003 Shenzhen, China

**Keywords:** Neural circuits, Social behaviour

Correction to: *Nature Communications* 10.1038/s41467-020-19102-3, published online 22 October 2020

The original version of this Article contains an error in the spelling of the name Dr. Hongtao Qin, which is incorrectly given as Dr. Hongtao Qing in the Acknowledgements section. This has now been corrected in both the PDF and HTML versions of the Article.

