# Supplementary material for: Social attraction in Drosophila is regulated by the mushroom body and serotonergic system
Source: Nat Commun. 2020 Oct 22;11:5350. doi: 10.1038/s41467-020-19102-3 (PMC7582864; doi:10.1038/s41467-020-19102-3)
Supplement: Supplementary file 1 — Supplementary Information [file 41467_2020_19102_MOESM1_ESM.pdf]

Supplementary Information

Social attraction in *Drosophila* is regulated by the mushroom body and serotonergic system

Sun *et al*.

Supplementary Figures 1-19

Supplementary Tables 1-3

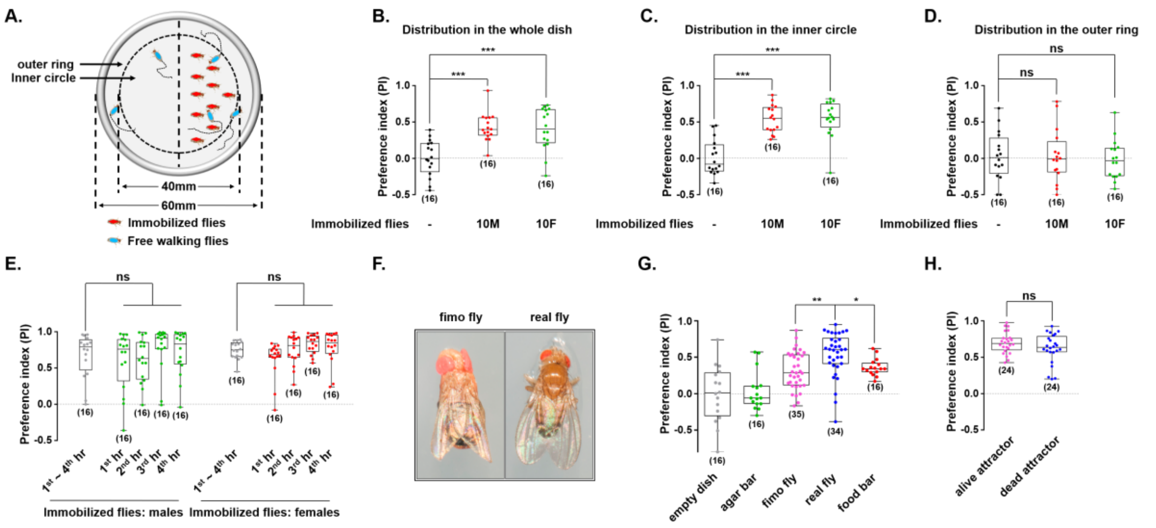

Supplementary Figure 1. Social approach behaviour of *Canton S* flies, related to Fig. 1. (A) Schematic illustrating the division of the observation field into the empty side (left) and the fly-containing side (right) by a vertical dotted line. The arena was further divided into an inner circle and an outer ring. (B–D) Comparing the distributions of free-walking flies in different areas: the whole arena, including both inner circle and outer ring (B), the inner circle (C), and the outer ring (D). (E) No significant changes in social attraction during a 4-hour observation period. (F) Pictures of a handmade Fimo fly (clay model of a fly with wings from a real fly F-a) and a real fly (F-b). Both were glued to the agar surface. (G) Effects of different attractors on social approach behaviour. Agar bars and food bars are cuboid-shaped bars made of agar or food. All of the attractors had the same physical dimensions as real flies. (H) Comparing the attractiveness of living CS attractor flies and dead CS attractor flies. Results are presented as a box and whisker plot; whiskers indicate the minimum and maximum, the box includes 25th–75th percentile, and the line in the box indicates the median of the data set. Analyzed numbers (n) from biologically independent samples are showed below each graph. Statistical analysis: one-way ANOVA followed by Dunnett's test for multiple comparisons (B–E and G) were used for most comparisons, and t-tests were used for comparisons of only two groups (H). ns: $P > 0.05$, ***: $P < 0.001$.

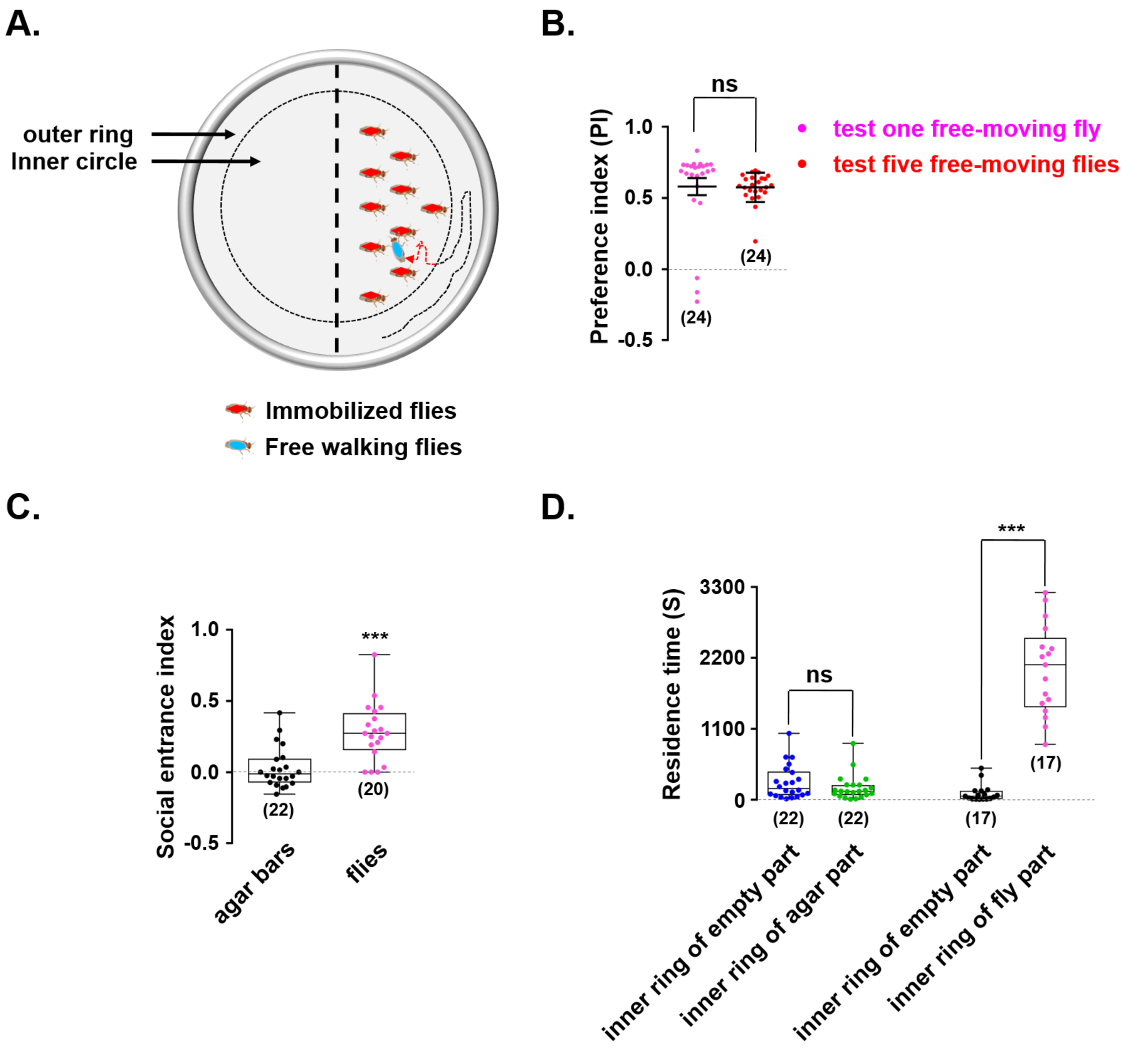

Supplementary Figure 2 Analysing social approach behaviour of single flies via video tracking, related to Fig. 1. (A) Schematic illustrating the features quantified in the behavioural analysis of single free-walking flies. (B) Varying the numbers of free-moving flies to one subject fly vs. five subject flies did not impact on social approach behaviour. (C) Comparing the entrance index of single flies when exploring the arena containing either agar bars or real flies. (D) Comparing the total residence times of single free-walking flies on the two sides of the inner ring. Results are presented as a scatter plot; error bars denote means ± SEM (B) and a box and whisker plot; the whiskers indicate the minimum and maximum, the box includes the 25th–75th percentile, and the line in the box indicates the median of the data set (C and D). Analyzed numbers (n) from

biologically independent samples are showed below each graph. Statistical analysis: unpaired t-test. ns: $P > 0.05$, ***: $P < 0.001$.

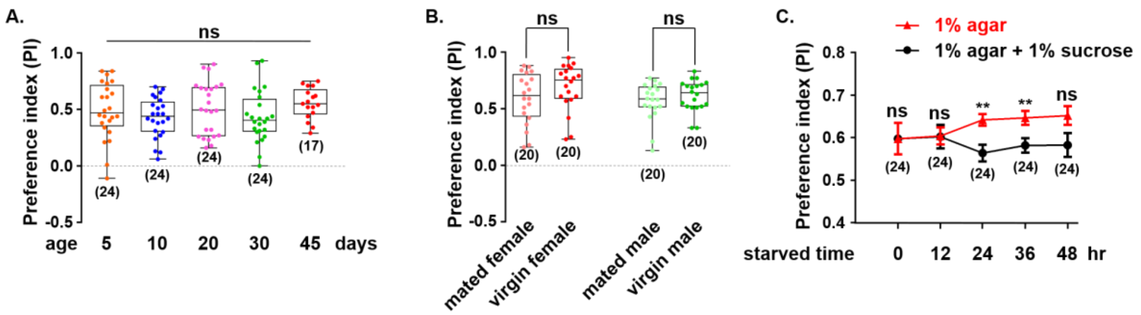

Supplementary Figure 3. Effects of physiological status on social approach behaviour, related to Fig. 1. (A) The age of the attractor flies had no influence on social attraction. (B) The sex and mating status of the attractor flies did not affect the social approach behaviour of wild type *CS* flies. (C) Hungry flies exhibited an increased tendency to approach attractor flies (not starved). Starved female flies were kept on 1% agar, while fed flies were kept on 1% agar and 1% sucrose for the indicated number of hours before the test. Results are presented as a box and whisker plot; the whiskers indicate the minimum and maximum, the box includes the 25th–75th percentile, and the line in the box indicates the median of the data set. Analyzed numbers (n) from biologically independent samples are showed below each graph. Statistical analysis: unpaired t-test (B and C) and one-way ANOVA followed by Tukey's test (A). ns: P > 0.05, **: P < 0.01.

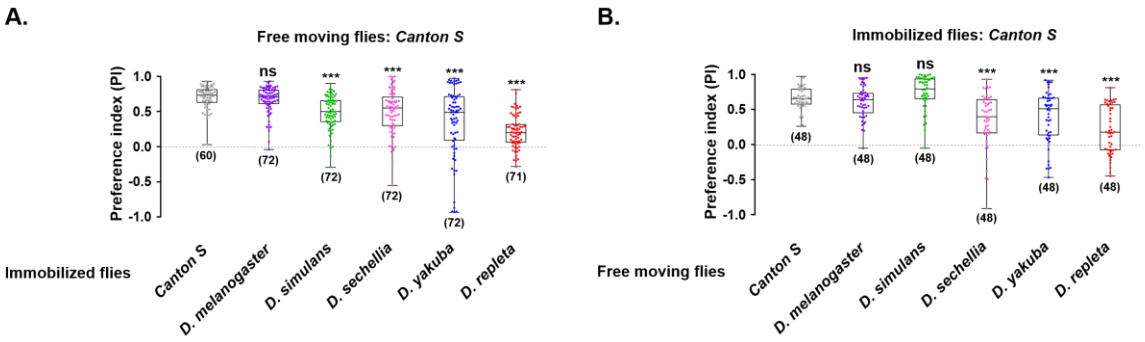

Supplementary Figure 4. Surveying social attraction in different species of wild flies, related to Fig. 1. (A) Testing the attractiveness of *D. melanogaster*, *D. simulans*, *D. sechellia*, *D. yakuba*, and *D. repleta* toward free-moving female *CS* flies. (B) Immobilized *CS* exhibited varying degrees of attraction toward other *Drosophilids*. Results are presented as a box and whisker plot; the whiskers indicate the minimum and maximum, the box includes the 25th–75th percentile, and the line in the box indicates the median of the data set. Analyzed numbers (n) from biologically independent samples are showed below each graph. Statistical analysis: one-way ANOVA followed by Dunnett's test was used for comparison of all boxes vs. control box. ns: $P > 0.05$, ***: $P < 0.001$.

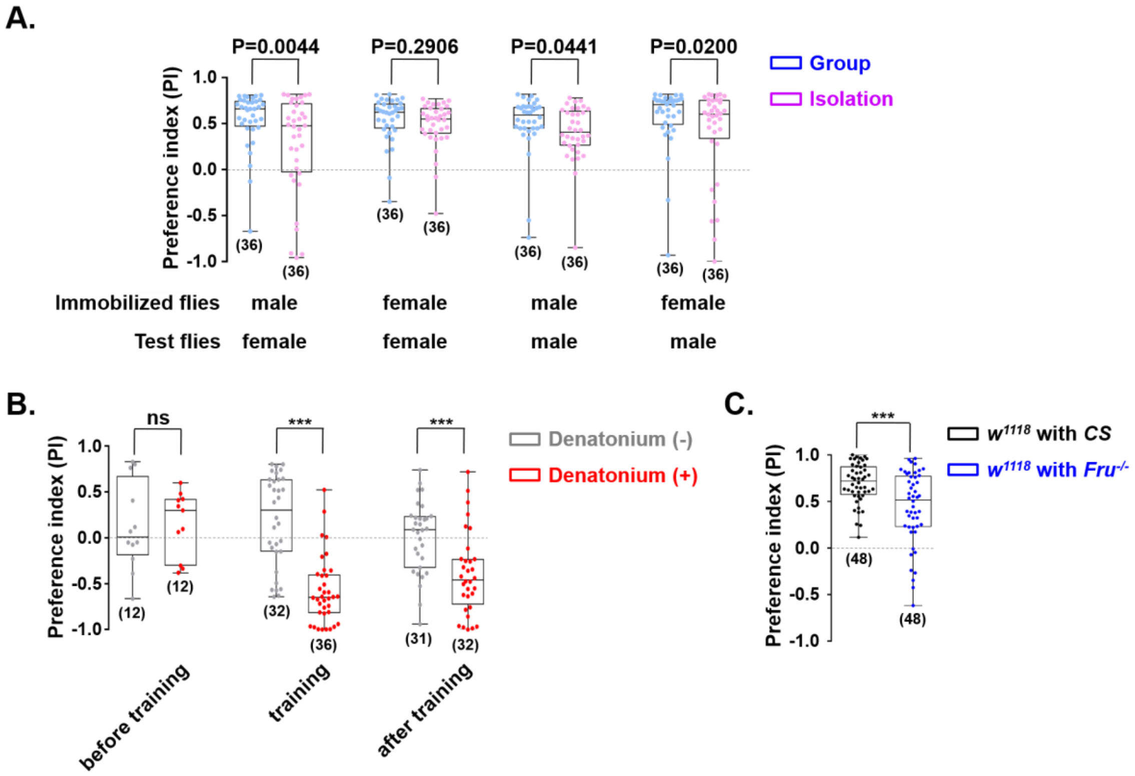

Supplementary Figure 5. Social experience influences social approach behaviour, related to Fig. 1. (A) Comparison of social motivation between singly-reared flies (isolation, purple) and group-reared flies (group, blue). (B) Wild type flies learned to associate social cues from attractor flies with punishment. Denatonium is a bitter compound to fruit flies. (C) Male flies being courted errantly by *fruitless* males (w^{1118} with $Fru^{-/-}$) for 1 day exhibited lower social motivation than control males, which were kept with normal males (w^{1118} with *CS*) for the same period. Results are presented as a box and whisker plot; the whiskers indicate the minimum and maximum, the box includes the 25th–75th percentile, and the line in the box indicates the median of the data set. Analyzed numbers (n) from biologically independent samples are showed below each graph. Statistical analysis: paired t-test (A) and unpaired t-test (B and C). ns: P > 0.05, ***: P < 0.001.

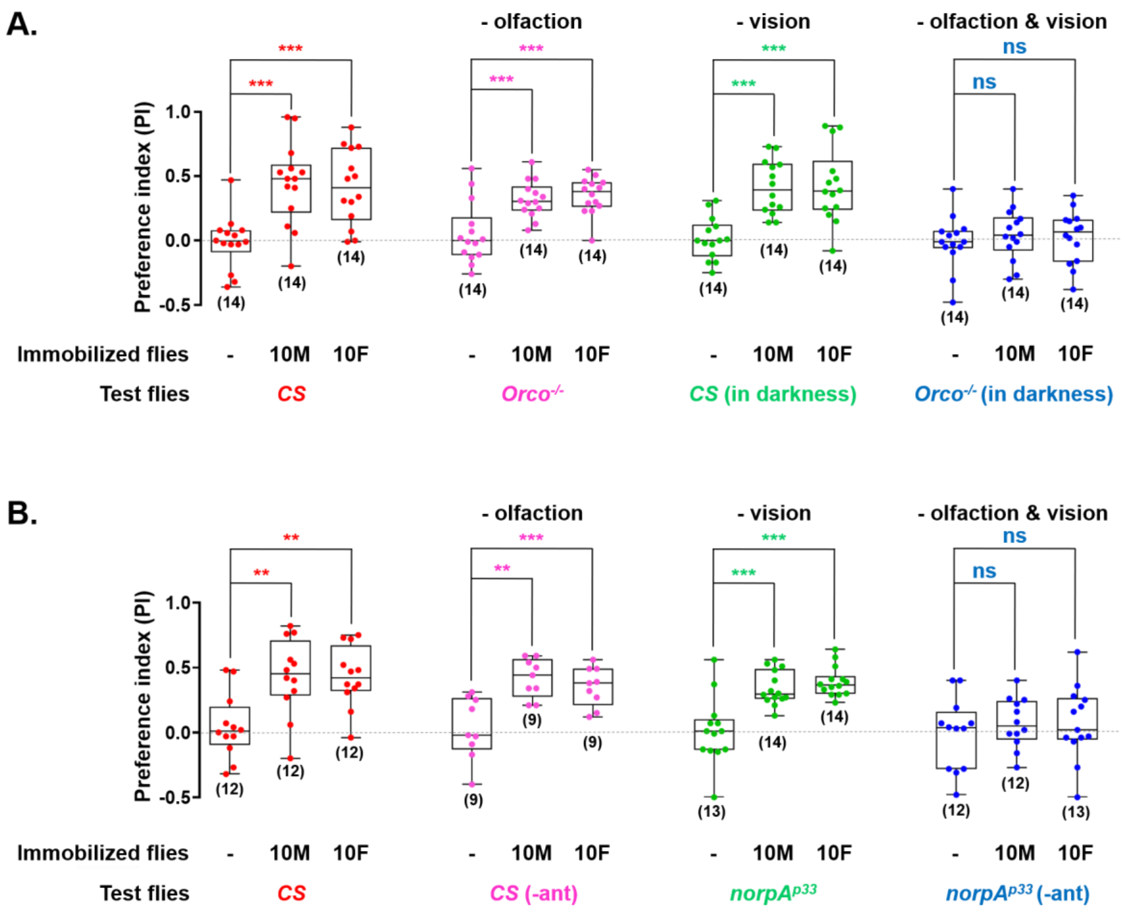

Supplementary Figure 6. Redundant sensory inputs for social attraction, related to Fig. 2. (A) Removing olfactory and visual inputs simultaneously by testing free-walking *Orco*$^{-/-}$ in the dark eliminated social approach responses to normal attractor flies. (B) Blocking olfactory and visual inputs together by testing the free-walking *norpA*^{p33} mutants without antennae abolished social approach behaviour. The results are presented as a box and whisker plot; the whiskers indicate the minimum and maximum, the box includes the 25th–75th percentile, and the line in the box indicates the median of the data set. Analyzed numbers (n) from biologically independent samples are showed below each graph. Statistical analysis: one-way ANOVA followed by Dunnett's test: comparison of all boxes vs. control box. ns: $P > 0.05$, **: $P < 0.01$, ***: $P < 0.001$.

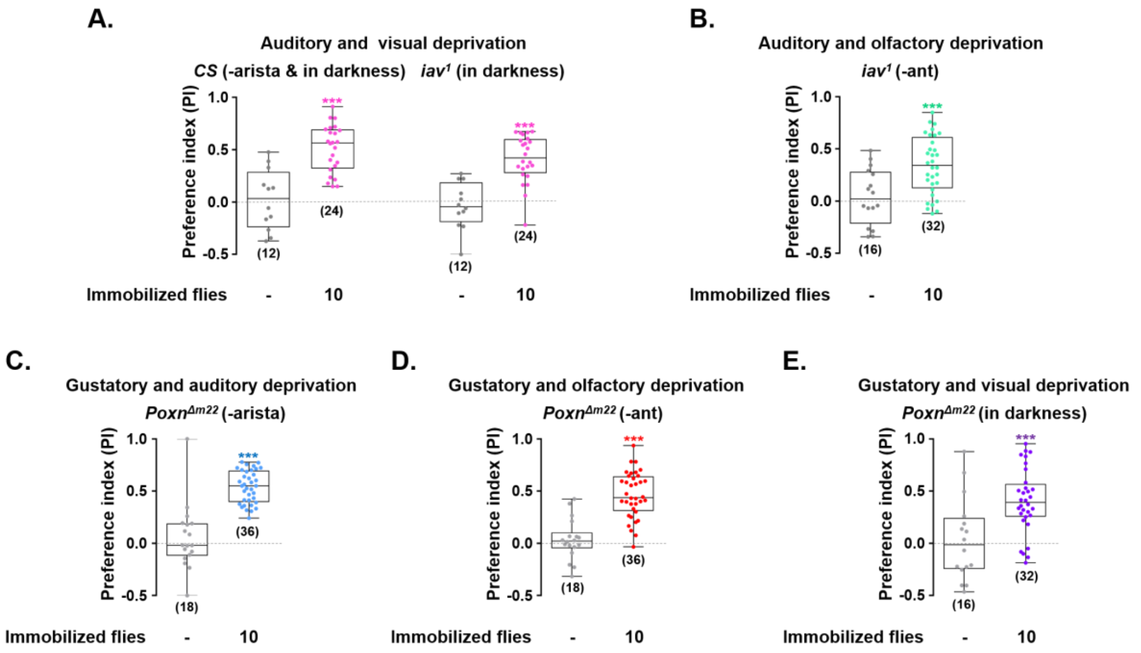

Supplementary Figure 7. Testing the effects of removing multiple sensory modalities on social approach behaviours, related to Fig. 2. (A) Evaluating the effects of depriving both auditory and visual senses. Free-walking *Canton S* with arista surgically removed and *iav*$^{-/-}$ mutants were tested in darkness. (B) Combined deprivation of auditory and olfactory senses had no impact on social approach behaviour. Free-walking *iav*$^{-/-}$ mutants with antennae surgically removed were tested. (C) Flies still exhibited normal social approach behaviour after the deprivation of both gustatory and auditory senses. Free-walking flies were *Poxn*$^{\Delta m22}$ mutants with arista surgically removed. (D) No effects were found in flies deprived of both gustatory and olfactory senses. Free-walking flies were *Poxn*$^{\Delta m22}$ mutants with antennae surgically removed. (E) Normal levels of social approach were found in flies deprived of both gustatory and visual senses. Free-walking *Poxn*$^{\Delta m22}$ mutants were tested in the dark. Results are presented as a box and whisker plot; the whiskers indicate the minimum and maximum, the box includes the 25th–75th percentile, and the line in the box indicates the median of the data set. Analyzed numbers (n) from biologically independent samples are showed below each graph. Statistical analysis: unpaired t-test. ***: $P < 0.001$.

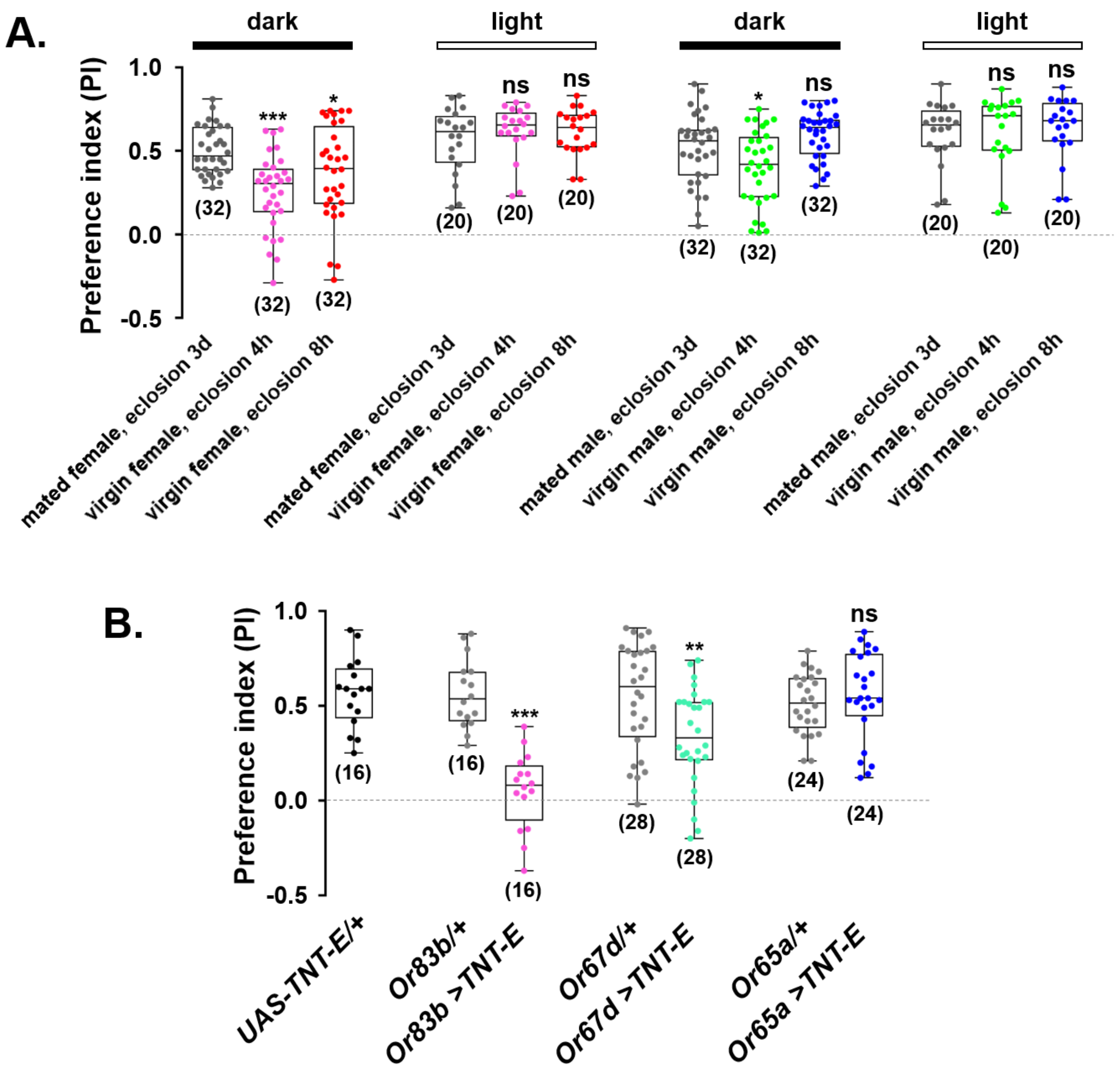

Supplementary Figure 8. cVA plays a partial role in mediating social approach behaviour. (A) Quantification of *Canton S* female flies attracted by various attractor flies: virgin females (eclosion for 4 hr: pink; eclosion for 8 hr: red); mated females (light grey), virgin males (eclosion for 4 hr: green; eclosion for 8 hr: blue), or mated males (dark grey). The bars on top of the bars indicated the test conditions: in the dark (black) and in the light (white). (B) Blocking *Or76d-GAL4* labelled olfactory receptor neurons impaired social motivation. Results are presented as a box and whisker plot; the whiskers indicate the minimum and maximum, the box includes the 25th–75th percentile, and the line in the box indicates the median of the data set. Analyzed numbers (n) from biologically independent samples are showed below each graph. Statistical analysis: one-way

ANOVA followed by Dunnett's test for multiple comparisons (A) were used for most comparisons, and t-tests were used for comparisons of only two groups (B). ns: $P > 0.05$, *: $P < 0.05$, **: $P < 0.01$, ***: $P < 0.001$.

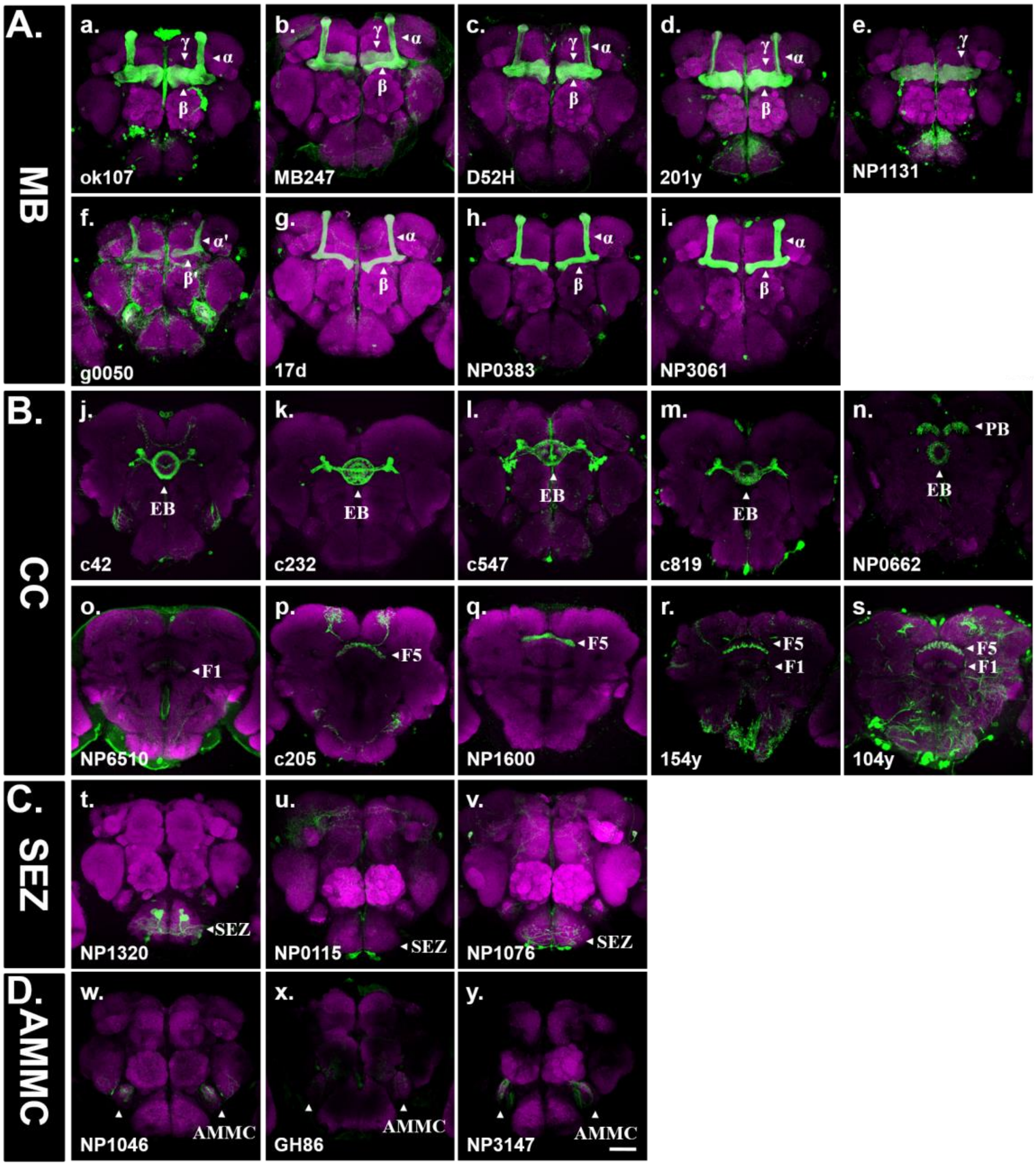

Supplementary Figure 9. Expression patterns of the GAL4 drivers used for inactivating neurons in major brain regions, related to Fig. 3. Expression patterns were visualized by crossing to *UAS-CD8::GFP*. The neuropil was counterstained with antibodies against nc82 (red). For each panel, a projection of a confocal stack of corresponding brain regions of a female fly is shown. (A) Expression patterns of mushroom body-Gal4 lines: ok107 (a), MB247 (b), D52H (c), 201y (d), NP1131 (e), g0050 (f), 17d (g), NP0383 (h), and NP3061 (i). The white arrowhead indicates three

major neuronal subregions of mushroom bodies: α/β, α'/β', and γ lobe. ok107, MB247, D52H, and 201y label all three KC neurons (α/β, α'/β', and γ neurons); 17d, NP0383, and NP3061 label only α/β neurons; g0050 labels only α'/β' neurons; NP1131 labels only γ neurons. (B) Expression patterns of central complex-Gal4 lines: c42 (j), c232 (k), c547 (l), c819 (m), NP0662 (n), NP6510 (o), c205 (p), NP1600 (q), 154y (r), and 104y (s). The white arrowheads indicated three major neuronal subregions of the central complex (ellipsoid body, fan-shaped body, and protocerebral bridge). c232 labels R3/R4d neurons of the ellipsoid body; c42, c547, and c819 label R2/R4m neurons of the ellipsoid body; NP0662 labels neurons of the ellipsoid body and protocerebral bridge; NP6510 label only F1 neurons of the fan-shaped body; NP1600 and c205 label only F5 neurons of the fan-shaped body; 154y and 104y label both F1 and F5 neurons of the fan-shaped body. (C) Expression patterns of suboesophageal ganglia related Gal4 lines: NP1320 (t), NP0115 (u), and NP1076 (v). (D) Expression patterns of AMMC-Gal4 lines: NP1046 (w), GH86 (x), and NP3147 (y). The scale bar is 50 μm.

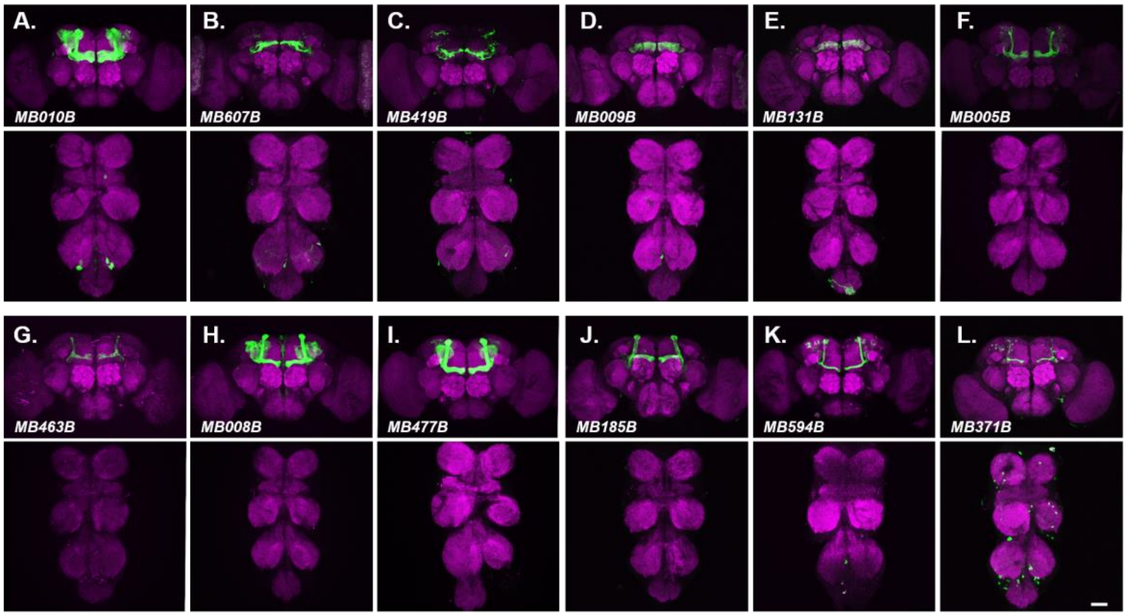

Supplementary Figure 10. Expression patterns of the GAL4 drivers used for silencing specific KC neurons. Expression patterns (green) were visualized by crossing each driver with UAS-CD8::GFP. The neuropil was counterstained with antibodies against nc82 (magenta). For each panel, projections of a confocal stack of the brain and the VNC region of a female fly were shown. (A–E) Expression patterns of KCγ-Gal4 lines. (F and G) Expression patterns of KCα′/β′-Gal4 lines. (H–L) Expression patterns of KCα/β-Gal4 lines. Scale bar = 50 µm.

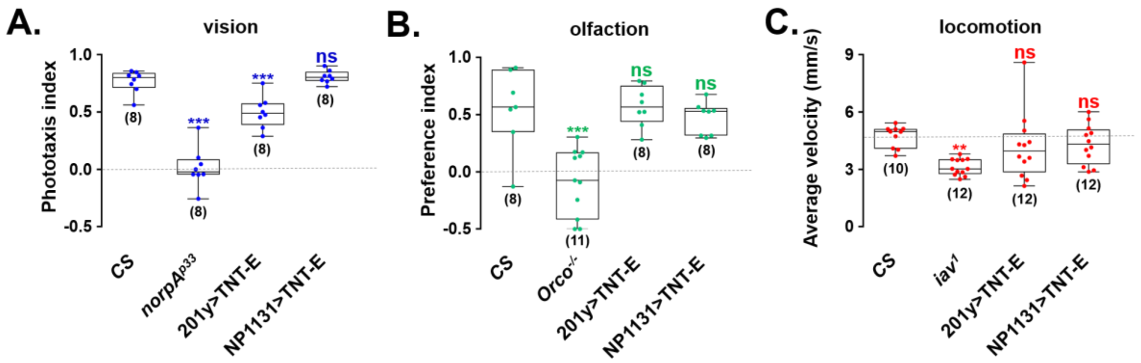

Supplementary Figure 11. Effects of silencing KC$_\gamma$ neurons on olfaction, vision, and locomotion ability, related to Fig. 3. The KC$_\gamma$ neurons were silenced via expressing TNT in 201y- or NP1131-labeled neurons. For each test, *Canton-S* and a related mutant served as negative and positive controls, respectively. (A) Flies displayed normal phototaxis ability after blocking KC$_\gamma$ neurons with TNT in NP1131 labelled neurons. (B) Flies with silenced KC$_\gamma$ neurons still exhibited normal preferences toward an attractive odour (apple cider vinegar). (C) Flies with silenced KC$_\gamma$ neurons exhibited a similar locomotion speed to *Canton S*. Results are presented as a box and whisker plot; the whiskers indicate the minimum and maximum, the box includes the 25th–75th percentile, and the line in the box indicates the median of the data set. Analyzed numbers (n) from biologically independent samples are showed below each graph. Statistical analysis: one-way ANOVA followed by Dunnett's test were used for comparison of all boxes vs. control box. ns: P > 0.05, **: P < 0.01, ***: P < 0.001.

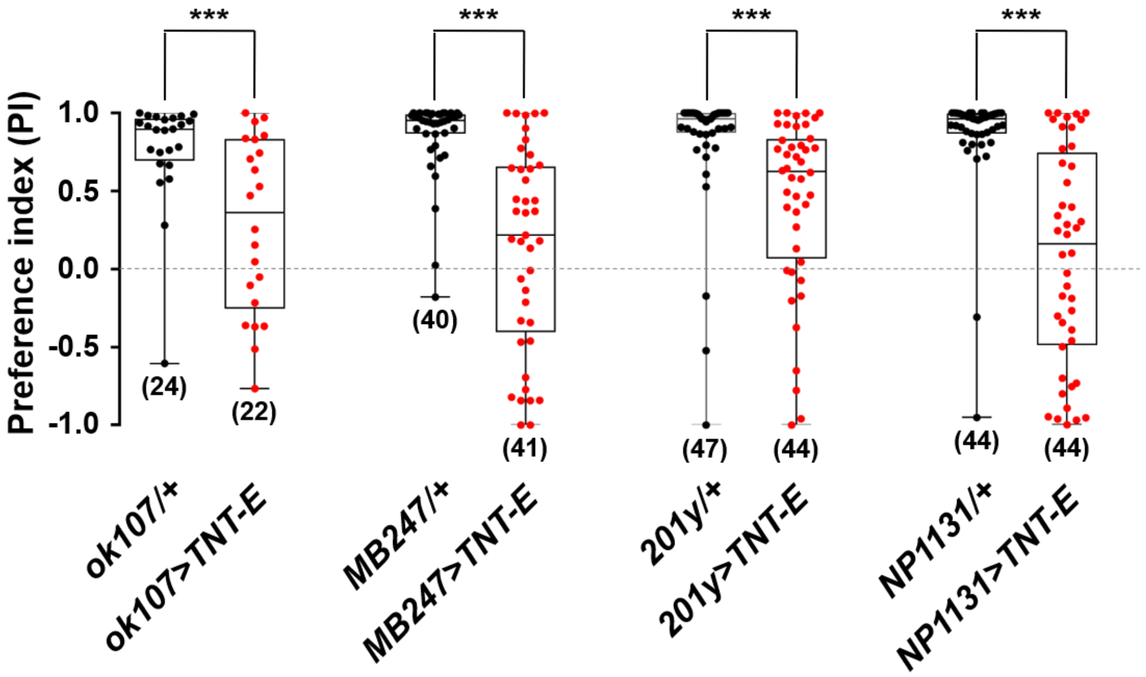

Supplementary Figure 12. KCγ neurons are necessary for the motivation of approaching conspecifics, related to Fig 3. The social approach of single flies was strongly decreased when expressing TNT in neurons labeled by ok107, MB247, 201y, and NP1131. Results are presented as a box and whisker plot, whiskers mark minimum and maximum, box includes 25th – 75th percentile, and the line in box indicates median of the data set. Analyzed numbers (n) from biologically independent samples are showed below each graph. Statistics: unpaired t-test. ***: P <0.001.

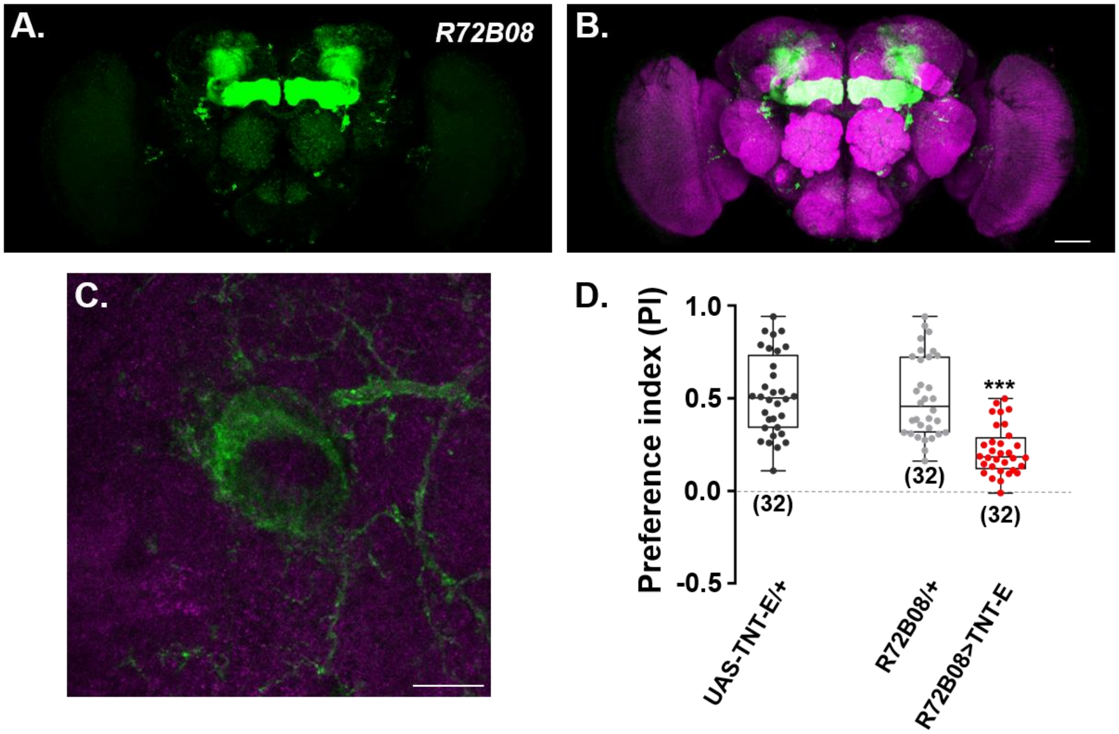

Supplementary Figure 13. Blocking *R72B08-Gal4* labeled neurons dramatically decreases the social approach level, related to Fig 4. Specific inactivation of the KC_{γ} neurons dramatically decreased the social approach level. (A and B) Expression patterns of *R72B08-Gal4* in the MB region were visualized by mCD8::GFP (green). Scale bars = 50 μm. (C) A cross-section of the peduncle of *R72B08>GFP*. Scale bars = 10 μm. (D) Blocking neurons labeled by *R72B08-Gal4* with TNT strongly impacted social affiliation. Results are presented as a box and whisker plot, whiskers mark minimum and maximum, box includes 25th – 75th percentile, and the line in box indicates median of the data set. Analyzed numbers (n) from biologically independent samples are showed below each graph. Statistics: unpaired t-test. ***: $P < 0.001$.

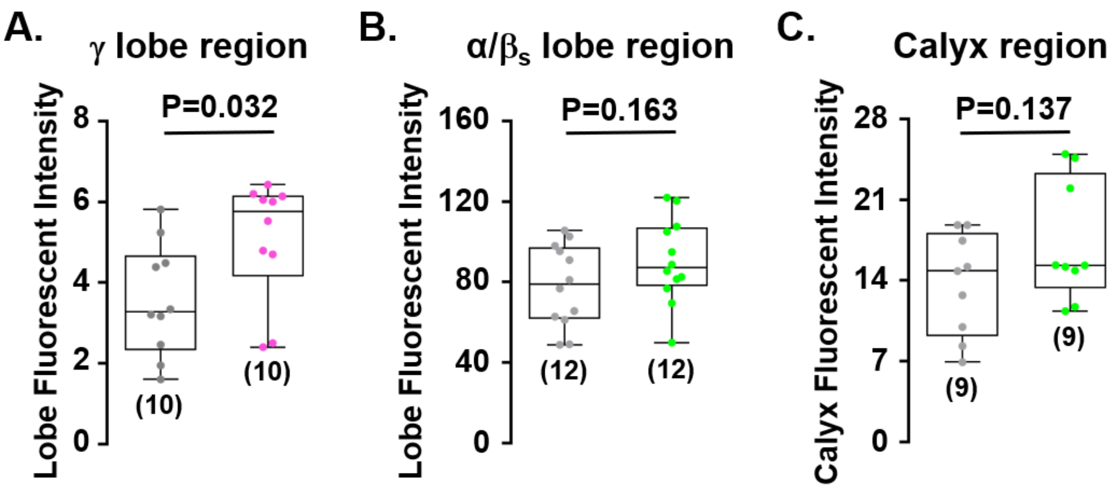

Supplementary Figure 14. The effects of social experience on the activity states of different KC neurons, related to Fig. 4. (A) The fluorescent intensities via the CaLexA method were compared between the group-reared flies and singly-reared flies in the γ lobe region. Confocal images of KC_{γ} neurons in flies bearing *R72B08-GAL4*, *UAS-mLexA-VP16-NFAT*, *LexAop-CD2-GFP*, and *LexAop-CD8-GFP-2A-CD8-GFP* transgenes were analysed. (B and C) The fluorescent intensities via the CaLexA method were compared between the group-reared flies and singly-reared flies in the α/β lobe region (B) and the calyx regions (C). Confocal images of $KC_{\alpha/\beta}$ neurons in flies bearing *NP3061-Gal4*, *UAS-mLexA-VP16-NFAT*, *LexAop-CD2-GFP*, and *LexAop-CD8-GFP-2A-CD8-GFP* transgenes were analysed. Results are presented as a box and whisker plot; the whiskers indicate the minimum and maximum, the box includes the 25th–75th percentile, and the line in the box indicates the median of the data set. Analyzed numbers (n) from biologically independent samples are showed below each graph. Statistical analysis: unpaired t-test.

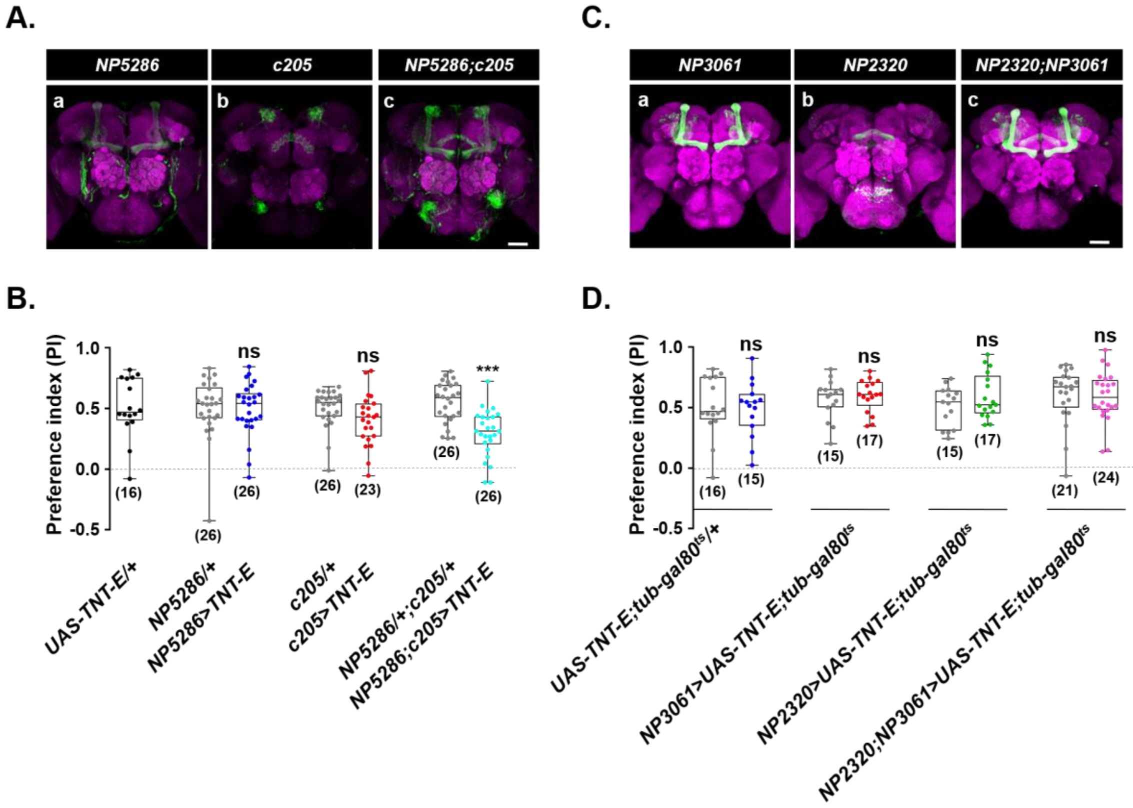

Supplementary Figure 15. Effects of simultaneously silencing F5 and MB neurons with TNT, related to Fig. 5. (A) Expression patterns (green) of NP5286 (a), c205 (b), and NP5286; c205 (c). (B) Inactivation of α/β surface neurons (NP5286) and F5 neurons (c205) simultaneously resulted in a strong reduction in social approach behaviour, while inactivation of either population alone did not affect social approach behaviour. (C) Expression patterns of NP3061 (a), NP2320 (b), and NP3061; NP2320 (c). (D) Inactivation of α/β surface neurons (NP3061) and large interneurons of the fan-shaped body (NP2320) simultaneously had no effect on social approach behaviour. The *tub-Gal80*^{ts} transgene and high-temperature were used to bypass developmental defects. Magenta indicates neuropil counterstaining with the antibody against nc82. The scale bar is 50 μm. Results are presented as a box and whisker plot; the whiskers indicate the minimum and maximum, the box includes the 25th–75th percentile, and the line in the box indicates the median of the data set. Analyzed numbers (n) from biologically independent samples are showed below each graph. Statistical analysis: unpaired t-test. ns: $P > 0.05$, ***: $P < 0.001$.

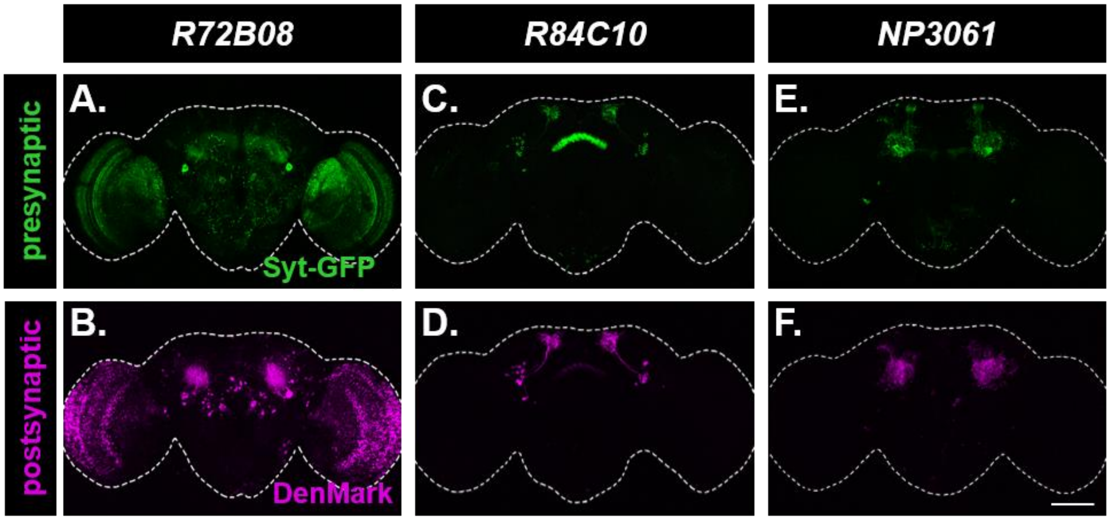

Supplementary Figure 16. Visualization of the pre- and postsynaptic structures of neurons labeled by R72B08, R84C10, and NP3061, related to Fig 5. The brains were outlined by white dotted lines. Presynaptic structures were revealed by expressing *UAS-syt.eGFP* in R72B08 (A), R84C10 (C), and NP3061 (E) labelled neurons. Postsynaptic structures were visualized by expressing *UAS-Denmark* in R72B08 (B), R84C10 (D), and NP3061 (F) labelled neurons. Scale bars = 50 μm.

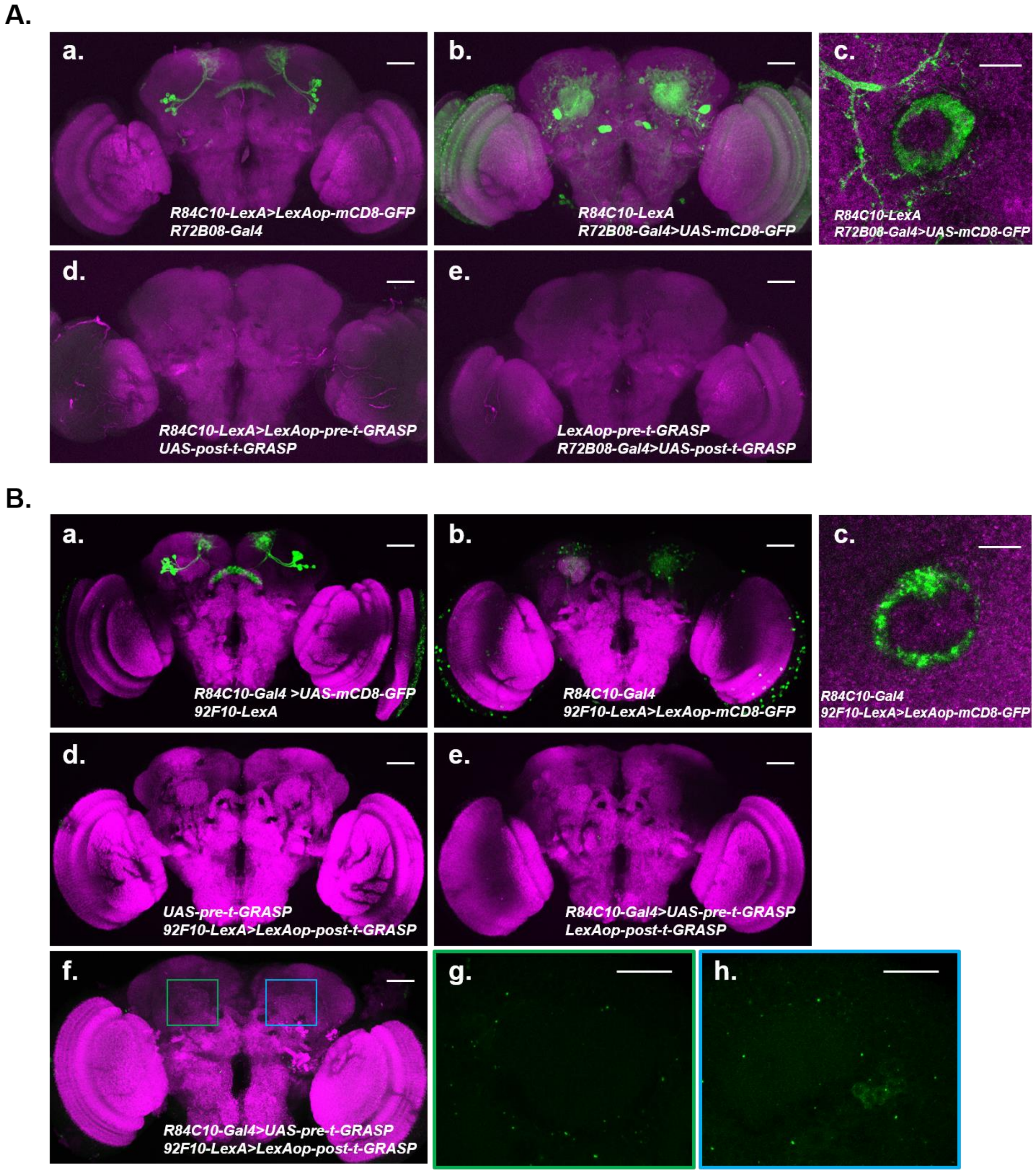

Supplementary Figure 17. Visualization of synaptic connections between KCγ neurons and F5 neurons in the calyx region, related to Fig. 5. (A) The genetic elements used in t-GRASP experiments for testing connectivity between F5 neurons (*LexA*-labelled) and KC_{γ} neurons (*Gal4*-labeled). Expression patterns of *R84C10-LexA* (A-a) and *R72B08-GAL4* (A-b) in the presence of the other driver. (A-c) A cross-section of the peduncle in a fly with the same genotype as (A-b). (A-d and A-e) GFP signals were

not detected in two negative controls for t-GRASP analysis. Neither *R84C10-LexA* driver (A-d) nor *R72B08-GAL4* driver (A-e) alone was sufficient to generate GFP signals. (B) Another set of genetic elements used in t-GRASP experiments for testing connectivity between F5 neurons (*Gal4*-labeled) and KC_{γ} neurons (*LexA*-labelled). *R84C10-GAL4* (B-a) and *92F10-LexA* (B-b) drove the expression of full-length GFP, in the presence of the other driver. (B-c) A cross-section of the peduncle in a fly with the same genotype as in (B-b). (B-d and B-e) GFP signals were not detected in two negative controls for t-GRASP analysis. Neither *92F10-LexA* driver (B-d) nor *R84C10-GAL4* driver (B-e) alone was sufficient to generate GFP signals. (B-f) The contact sites between KC_{γ} neurons (*92F10-LexA*) and F5 neurons (*R84C10-Gal4*) in the calyx region (coloured box) were visualized by t-GRASP. (B-g and B-h) Magnified views of t-GRASP signals in the boxed regions of (B-f). The neuropil was counterstained with antibodies against nc82 (magenta). Scale bars = 50 μm, except in (A-c, B-c, B-g, and B-h), where scale bars = 10 μm.

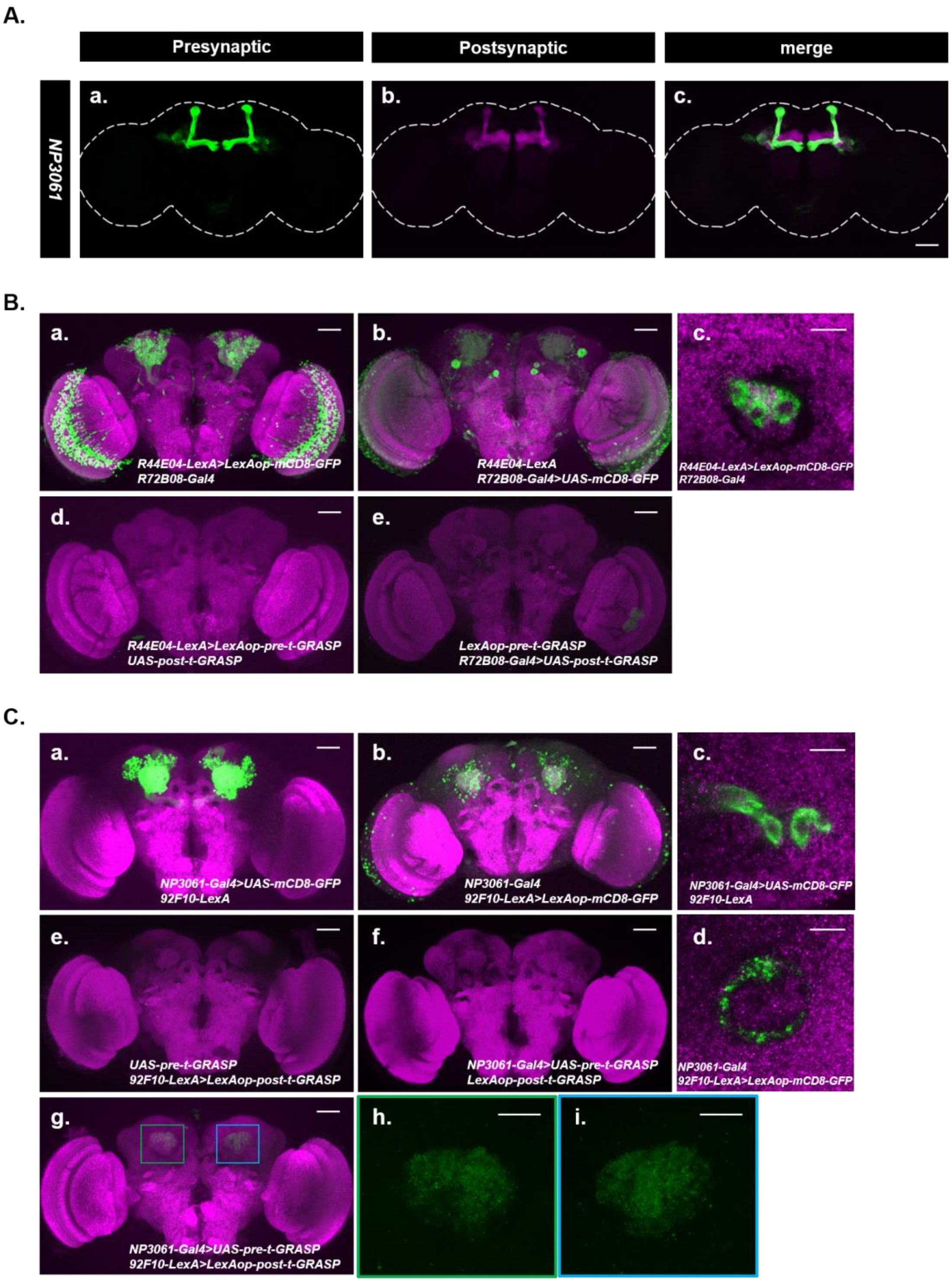

Supplementary Figure 18. Visualization of synaptic connections between KCα/β neurons and KCγ neurons in the calyx region, related to Fig. 5. (A) In flies bearing the trans-Tango components, *NP3061-Gal4* drove the presynaptic expressions of ligand

and myrGFP in the α/β lobes of MB (A-a, green) and also generated postsynaptic mtdTomato signals in α/β lobes and γ lobes of MB (A-b, magenta). The brains were outlined by white dotted lines. (B) The genetic elements used in t-GRASP experiments for testing connectivity between $KC_{α/β}$ neurons (*LexA*-labelled) and $KC_γ$ neurons (*Gal4*-labeled). Expression patterns of *R44E04-LexA* ($KC_{α/β}$ neurons, B-a) and *R72B08-GAL4* ($KC_γ$ neurons, B-b) in brains as visualized by GFP signals. (B-c) A cross-section of the peduncle in a fly with the same genotype as (B-a). (B-d and B-e) GFP signals were not detected in two negative controls for t-GRASP analysis. Neither *R44E04-LexA* driver (B-d) nor *R72B08-GAL4* driver (B-e) alone was sufficient to generate GFP signals. (C) Another set of genetic elements used in t-GRASP experiments for testing connectivity between $KC_{α/β}$ neurons (*Gal4*-labeled) and $KC_γ$ neurons (*LexA*-labelled). *NP3061-GAL4* ($KC_{α/β}$ neurons, C-a) and *92F10-LexA* ($KC_γ$ neurons, C-b) drove the expression of full-length GFP, in the presence of the other driver. (C-c) A cross-section of the peduncle in a fly with the same genotype as (C-a). (C-d) A cross-section of the peduncle in a fly with the same genotype as (C-b). (C-e and C-f) GFP signals were not detected in two negative controls for t-GRASP analysis. (C-g) The contact sites between $KC_{α/β}$ neurons (*NP3061-GAL4*) and $KC_γ$ neurons (*92F10-LexA*) in the calyx region (coloured box) were visualized by t-GRASP. (C-h and C-l) Magnified views of t-GRASP signals in the boxed regions in (C-g). The neuropil was counterstained with antibodies against nc82 (magenta). Scale bars = 50 μm, except in (B-c, C-c, and C-d: 25 μm) and in (C-h and C-l: 10 μm).

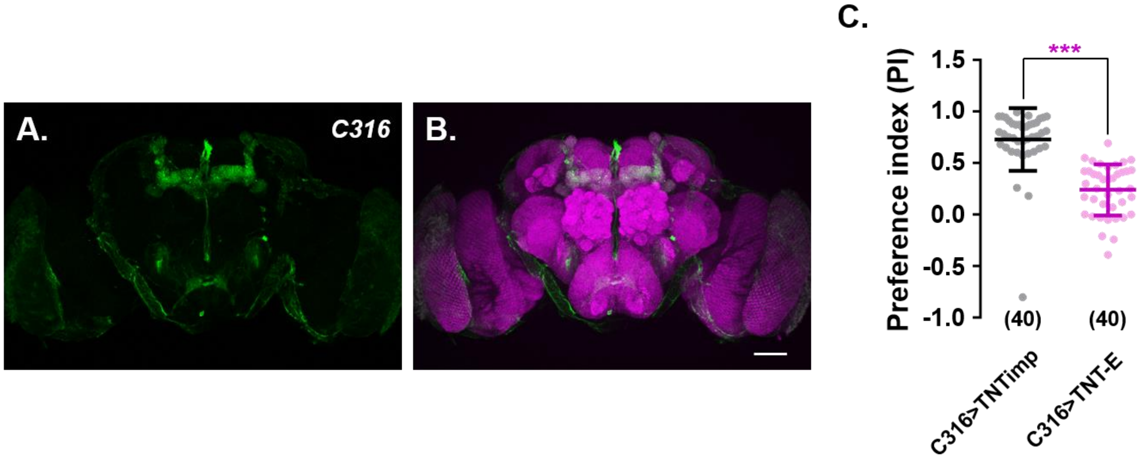

Supplementary Figure 19 Blocking DPM neurons greatly decreases social approach behaviour, related to Fig. 6. (A and B) Expression patterns of *C316-Gal4* were visualized by mCD8::GFP (green). The neuropil was counterstained with antibodies against nc82 (magenta). Scale bar = 50 µm. (C) Silencing *C316-Gal4*-labeled serotoninergic neurons strongly suppressed social motivation. Results are presented as a scatter plot; error bars denote means ± SEM. Analyzed numbers (n) from biologically independent samples are showed below each graph. Statistical analysis: unpaired t-test. ***: P < 0.001.

Supplementary Table 1. Social attraction uses multiple sensory cues

Removed cure(s)	Immobilized flies	Test flies	Preference index
-	*Canton S*	*Canton S*	0.45±0.14
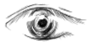	*Canton S*	*NorpA*^{p33}	0.50±0.15
	Canton S	In dark	0.48±0.16
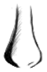	*Canton S*	*Orco*$^{-/-}$	0.33±0.19*
	Canton S	*CS*, -antenna	0.45±0.32
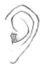	*Canton S*	*iav*^{1}	0.51±0.27
	Canton S	*CS*, -arista	0.65±0.18
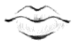	*Canton S*	*Poxn*$^{\Delta m22}$	0.49±0.35
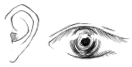	*Canton S*	*iav*^{1}, in dark	0.41±0.23
	Canton S	-arista, in dark	0.51±0.23
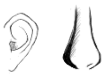	*Canton S*	*iav*^{1}, -arista	0.34±0.28
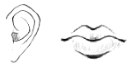	*Canton S*	*Poxn*$^{\Delta m22}$, -arista	0.54±0.16
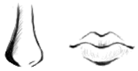	*Canton S*	*Poxn*$^{\Delta m22}$, -antenna	0.46±0.22
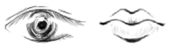	*Canton S*	*Poxn*$^{\Delta m22}$, in dark	0.40±0.31
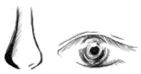	*Canton S*	*Orco*$^{-/-}$, in dark	0.03±0.20***
	Canton S	*CS*, -antenna, in dark	0.05±0.19***
	Canton S	*NorpA*^{p33}, -antenna	0.07±0.26***

Supplementary Table 2. γ lobe neurons are necessary for motivation of social approach

Fly	Fan-shaped body		Ellipsoid body		Protocerebral brigdge	Mushroom body			SEZ	AM MC	PI
	F1	F5	R3/R4d	R2/R4m		α/β	α'/β'	γ			
ok107						○	○	●			↓
MB247						○		●			↓
201y						○		●	○		↓
D52H						○		●			↓
NP1131				○			○	●	○		↓
17d						○					—
NP0383						○					—
NP3061						○					—
g0050		○					○				—
c42		○		○						○	—
c232			○								—
c547				○							—
c819				○							—
NP6510	○										—
104y	○	○									—
c205		○							○	○	—
NP1600		○									—
154y	○	○									—
NP0662		○			○						—
NP1320									○		—
NP115									○		—
NP1076									○		—
NP1046										○	—
GH86										○	—
NP3147										○	—

Supplementary Table 3. Key resources table

Reagent type (species) or resource	Designation	Source or reference	Identifiers
Genetic reagent (*Drosophila melanogaster*)	*Canton-S*	Zhan et al., 2016 (doi: 10.1038/ncomms13633)	
Genetic reagent (*Drosophila melanogaster*)	*Oregon-R*	Bloomington *Drosophila* Stock Center	RRID: BDSC 6361
Genetic reagent (*Drosophila melanogaster*)	*Berlin-k*	Bloomington *Drosophila* Stock Center	RRID: BDSC 8522
Genetic reagent (*Drosophila melanogaster*)	w^{1118}	Bloomington *Drosophila* Stock Center	RRID: BDSC 5905
Genetic reagent (*Drosophila melanogaster*)	*yw*	Bloomington *Drosophila* Stock Center	RRID: BDSC 10209
Genetic reagent (*Drosophila melanogaster*)	*D. melanogaster*	Core Facility of Drosophila Resource and Technology, Shanghai Institute of Biochemistry and Cell Biology, Chinese Academy of Scieces	
Genetic reagent (*Drosophila melanogaster*)	*D. simulans*	Core Facility of Drosophila Resource and Technology, Shanghai Institute of Biochemistry and Cell Biology, Chinese Academy of Scieces	

Genetic reagent (*Drosophila melanogaster*)	*D. sechellia*	Core Facility of Drosophila Resource and Technology, Shanghai Institute of Biochemistry and Cell Biology, Chinese Academy of Scieces	
Genetic reagent (*Drosophila melanogaster*)	*D. yakuba*	Core Facility of Drosophila Resource and Technology, Shanghai Institute of Biochemistry and Cell Biology, Chinese Academy of Scieces	
Genetic reagent (*Drosophila melanogaster*)	*D. repleta*	Core Facility of Drosophila Resource and Technology, Shanghai Institute of Biochemistry and Cell Biology, Chinese Academy of Scieces	
Genetic reagent (*Drosophila melanogaster*)	fru^{LexA}	Bruce S. Baker lab, Janelia Farm Research Campus, Howard Hughes Medical Institute	
Genetic reagent (*Drosophila melanogaster*)	$fru^{4\text{-}40}$	Bruce S. Baker lab, Janelia Farm Research Campus, Howard Hughes Medical Institute	
Genetic reagent (*Drosophila melanogaster*)	$Orco^{-/-}$	Yi Rao lab, Peking University	
Genetic reagent (*Drosophila melanogaster*)	$norpA^{p33}$	Bloomington *Drosophila* Stock Center	RRID: BDSC 9047
Genetic reagent (*Drosophila melanogaster*)	iav^{1}	Yi Rao lab, Peking University	

Genetic reagent (*Drosophila melanogaster*)	*Poxn*$^{\Delta m22}$	Yi Rao lab,<br>Peking University
Genetic reagent (*Drosophila melanogaster*)	*UAS-TNT*	Aike Guo and Yan Li lab,<br>Institute of Biophysics,<br>Chinese Academy of Scieces
Genetic reagent (*Drosophila melanogaster*)	*UAS-TNT* (in Fig. S19)	Chuan Zhou lab,<br>Institute of Zoology,<br>Chinese Academy of Scieces
Genetic reagent (*Drosophila melanogaster*)	*UAS-TNTimp*	Chuan Zhou lab,<br>Institute of Zoology,<br>Chinese Academy of Scieces
Genetic reagent (*Drosophila melanogaster*)	*ok107*	Aike Guo and Yan Li lab,<br>Institute of Biophysics,<br>Chinese Academy of Scieces
Genetic reagent (*Drosophila melanogaster*)	*MB247*	Aike Guo and Yan Li lab,<br>Institute of Biophysics,<br>Chinese Academy of Scieces
Genetic reagent (*Drosophila melanogaster*)	*201y*	Aike Guo and Yan Li lab,<br>Institute of Biophysics,<br>Chinese Academy of Scieces
Genetic reagent (*Drosophila melanogaster*)	*NP1131*	Joshua Dubnau lab,<br>Cold Spring Harbor Laboratory

Genetic reagent (*Drosophila melanogaster*)	*D52H*		Joshua Dubnau lab,<br>Cold Spring Harbor Laboratory
Genetic reagent (*Drosophila melanogaster*)	*17d*		Aike Guo and Yan Li lab,<br>Institute of Biophysics,<br>Chinese Academy of Scieces
Genetic reagent (*Drosophila melanogaster*)	*NP0383*		Li Liu lab,<br>Institute of Biophysics,<br>Chinese Academy of Scieces
Genetic reagent (*Drosophila melanogaster*)	*NP3061*		Joshua Dubnau lab,<br>Cold Spring Harbor Laboratory
Genetic reagent (*Drosophila melanogaster*)	*g0050*		Joshua Dubnau lab,<br>Cold Spring Harbor Laboratory
Genetic reagent (*Drosophila melanogaster*)	*NP6649*		Li Liu lab,<br>Institute of Biophysics,<br>Chinese Academy of Scieces
Genetic reagent (*Drosophila melanogaster*)	*NP5286*		Yi Rao lab,<br>Peking University
Genetic reagent (*Drosophila melanogaster*)	*c232*		Li Liu lab,<br>Institute of Biophysics,<br>Chinese Academy of Scieces

Genetic reagent (*Drosophila melanogaster*)	*c819*	Li Liu lab, Institute of Biophysics, Chinese Academy of Scieces		
Genetic reagent (*Drosophila melanogaster*)	*c42*	Li Liu lab, Institute of Biophysics, Chinese Academy of Scieces		
Genetic reagent (*Drosophila melanogaster*)	*c547*	Li Liu lab, Institute of Biophysics, Chinese Academy of Scieces		
Genetic reagent (*Drosophila melanogaster*)	*NP6510*	Li Liu lab, Institute of Biophysics, Chinese Academy of Scieces		RRID: DGRC 113956
Genetic reagent (*Drosophila melanogaster*)	*104y*	Li Liu lab, Institute of Biophysics, Chinese Academy of Scieces		
Genetic reagent (*Drosophila melanogaster*)	*NP2320*	Li Liu lab, Institute of Biophysics, Chinese Academy of Scieces		RRID: DGRC 104157
Genetic reagent (*Drosophila melanogaster*)	*c205*	Li Liu lab, Institute of Biophysics, Chinese Academy of Scieces		
Genetic reagent (*Drosophila melanogaster*)	*NP1600*	Li Liu lab, Institute of Biophysics, Chinese Academy of Scieces		RRID: DGRC 112725

Genetic reagent (*Drosophila melanogaster*)	*154y*	Li Liu lab, Institute of Biophysics, Chinese Academy of Scieces	
Genetic reagent (*Drosophila melanogaster*)	*NP1320*	Li Liu lab, Institute of Biophysics, Chinese Academy of Scieces	RRID: DGRC 103985
Genetic reagent (*Drosophila melanogaster*)	*NP0115*	Li Liu lab, Institute of Biophysics, Chinese Academy of Scieces	RRID: DGRC 103529
Genetic reagent (*Drosophila melanogaster*)	*NP1076*	Li Liu lab, Institute of Biophysics, Chinese Academy of Scieces	RRID: DGRC 103876
Genetic reagent (*Drosophila melanogaster*)	*NP0662*	Li Liu lab, Institute of Biophysics, Chinese Academy of Scieces	RRID: DGRC 103701
Genetic reagent (*Drosophila melanogaster*)	*NP3147*	Li Liu lab, Institute of Biophysics, Chinese Academy of Scieces	RRID: DGRC 113126
Genetic reagent (*Drosophila melanogaster*)	*GH86*	Li Liu lab, Institute of Biophysics, Chinese Academy of Scieces	
Genetic reagent (*Drosophila melanogaster*)	*NP1046*	Li Liu lab, Institute of Biophysics, Chinese Academy of Scieces	RRID: DGRC 103867

Genetic reagent (*Drosophila melanogaster*)	*MB010B*	Yi Zhong Lab, Tsinghua University	RRID: BDSC 68293
Genetic reagent (*Drosophila melanogaster*)	*MB607B*	Yi Zhong Lab, Tsinghua University	RRID: BDSC 68256
Genetic reagent (*Drosophila melanogaster*)	*MB419B*	Yi Zhong Lab, Tsinghua University	RRID: BDSC 68323
Genetic reagent (*Drosophila melanogaster*)	*MB009B*	Yi Zhong Lab, Tsinghua University	RRID: BDSC 68292
Genetic reagent (*Drosophila melanogaster*)	*MB131B*	Yi Zhong Lab, Tsinghua University	RRID: BDSC 68265
Genetic reagent (*Drosophila melanogaster*)	*MB008B*	Hongtao Qin Lab, College of Biology, Hunan University	RRID: BDSC 68291
Genetic reagent (*Drosophila melanogaster*)	*MB477B*	Hongtao Qin Lab, College of Biology, Hunan University	RRID: BDSC 68328
Genetic reagent (*Drosophila melanogaster*)	*MB185B*	Yi Zhong Lab, Tsinghua University	RRID: BDSC 68267

Genetic reagent (*Drosophila melanogaster*)	*MB594B*	Hongtao Qin Lab, College of Biology, Hunan University	RRID: BDSC 68255
Genetic reagent (*Drosophila melanogaster*)	*MB371B*	Yi Zhong Lab, Tsinghua University	RRID: BDSC 68383
Genetic reagent (*Drosophila melanogaster*)	*MB005B*	Yi Zhong Lab, Tsinghua University	RRID: BDSC 68306
Genetic reagent (*Drosophila melanogaster*)	*MB463B*	Hongtao Qin Lab, College of Biology, Hunan University	RRID: BDSC 68370
Genetic reagent (*Drosophila melanogaster*)	*tub-GAL80*^{ts}	Aike Guo and Yan Li lab, Institute of Biophysics, Chinese Academy of Scieces	
Genetic reagent (*Drosophila melanogaster*)	*UAS-syt;DenMark*	Bloomington *Drosophila* Stock Center	RRID: BDSC 33064
Genetic reagent (*Drosophila melanogaster*)	*R72B08-Gal4*	Bloomington *Drosophila* Stock Center	RRID: BDSC 46669
Genetic reagent (*Drosophila melanogaster*)	*CaLexA*	Yi Rao lab, Peking University (doi: 10.3109/01677063.2011.642910)	

Genetic reagent (*Drosophila melanogaster*)	*trans-Tango*	Bloomington *Drosophila* Stock Center (doi: 10.1016/j.neuron.2017.10.011)	RRID: BDSC 77124
Genetic reagent (*Drosophila melanogaster*)	*UAS-CsChrimson*	Bloomington *Drosophila* Stock Center	RRID: BDSC 55136
Genetic reagent (*Drosophila melanogaster*)	UAS-mCD8::GFP	Zhan et al., 2016 (doi: 10.1038/ncomms13633)	
Genetic reagent (*Drosophila melanogaster*)	R84C10-Gal4	Li Liu lab, Institute of Biophysics, Chinese Academy of Scieces	RRID: BDSC 48378
Genetic reagent (*Drosophila melanogaster*)	*R84C10-LexA*	Li Liu lab, Institute of Biophysics, Chinese Academy of Scieces	RRID: BDSC 54339
Genetic reagent (*Drosophila melanogaster*)	*R44E04-LexA*	Yoshi Aso Lab, Janelia Farm Research Campus, Howard Hughes Medical Institute	RRID: BDSC 52736
Genetic reagent (*Drosophila melanogaster*)	*92F10-LexA*	Yoshi Aso Lab, Janelia Farm Research Campus, Howard Hughes Medical Institute	
Genetic reagent (*Drosophila melanogaster*)	*t-GRASP*	Bloomington *Drosophila* Stock Center	RRID: BDSC 79039 BDSC 79040

Genetic reagent (*Drosophila melanogaster*)	*TPH-Gal4*	Yi Rao lab,<br>Peking University	
Genetic reagent (*Drosophila melanogaster*)	*tsh-Gal80*	Aike Guo and Yan Li lab,<br>Institute of Biophysics,<br>Chinese Academy of Scieces	
Genetic reagent (*Drosophila melanogaster*)	*elav-Gal80*	Aike Guo and Yan Li lab,<br>Institute of Biophysics,<br>Chinese Academy of Scieces	
Genetic reagent (*Drosophila melanogaster*)	*5HT1B-Gal4 (III, HMS5)*	Yi Rao lab,<br>Peking University	
Genetic reagent (*Drosophila melanogaster*)	*UAS-5HT1B (II, HMS2)*	Yi Rao lab,<br>Peking University	
Genetic reagent (*Drosophila melanogaster*)	*UAS-5HT1B RNAi (II, HMS4)* in Fig. 6F & G	Yi Rao lab,<br>Peking University	
Genetic reagent (*Drosophila melanogaster*)	*UAS-5HT1B RNAi (III, HMS3)* in Fig. 6I	Yi Rao lab,<br>Peking University	
Genetic reagent (*Drosophila melanogaster*)	*elav-Gal4/+; UAS-Dcr2*	TsingHua Fly Center	RRID:<br>TB 00004

Genetic reagent (*Drosophila melanogaster*)	*UAS-5HT1A RNAi*	TsingHua Fly Center	RRID: TB 1216
Genetic reagent (*Drosophila melanogaster*)	*UAS-5HT2A RNAi*	TsingHua Fly Center	RRID: TB 2257
Genetic reagent (*Drosophila melanogaster*)	*UAS-5HT2B RNAi*	TsingHua Fly Center	RRID: TB 2078
Genetic reagent (*Drosophila melanogaster*)	*UAS-5HT7 RNAi*	TsingHua Fly Center	RRID: TB 0916
Genetic reagent (*Drosophila melanogaster*)	*MB-Gal80*	Aike Guo and Yan Li lab, Institute of Biophysics, Chinese Academy of Scieces	
Genetic reagent (*Drosophila melanogaster*)	*C316-Gal4*	Yulong Li lab, Peking University	
Antibody compound, drug	mouse anti-nc82	DSHB	CAT# AB_2314866
Antibody compound, drug	Rhodamine (TRITC) Conjugated Goat anti-Mouse IgG (H+L)	ZSGB-BIO	CAT# ZF-0313

Chemical compound, drug	All-trans-retinal	Sigma Aldrich	Cat #: R2500
Software, algorithm	*Prism 6*	GraphPad Prism https://www.graphpad.com/	RRID: SCR_002798
Software, algorithm	*MATLAB 2018a*	MathWorks, Natick, MA https://www.mathworks.com/products/matlab.html	RRID: SCR_006752
Software, algorithm	*Adobe Photoshop CC 2019*	Adobe https://www.adobe.com/	
Software, algorithm	*Fiji*	NIH https://fiji.sc/	RRID: SCR_002285
