# Supplementary material for: Social attraction in Drosophila is regulated by the mushroom body and serotonergic system
Source: Nat Commun. 2020 Oct 22;11:5350. doi: 10.1038/s41467-020-19102-3 (PMC7582864; doi:10.1038/s41467-020-19102-3)
Supplement: Supplementary file 3 — Description of Additional Supplementary Files [file 41467_2020_19102_MOESM3_ESM.pdf]

Description of Additional Supplementary Files

File Name: Supplementary Movie 1
Description: **Visualization of social approach behaviour and its quantification.** A sped-up video illustrates the procedure of analysing social approach behaviour. Relevant areas for analysis were indicated by coloured lines. The static background image (including the tethered attractor flies) was subtracted from each frame to extract the locations of free-walking flies. The distributions of flies over time were computed and plotted.
