# Supplementary material for: Social attraction in Drosophila is regulated by the mushroom body and serotonergic system
Source: Nat Commun. 2020 Oct 22;11:5350. doi: 10.1038/s41467-020-19102-3 (PMC7582864; doi:10.1038/s41467-020-19102-3)
Supplement: Supplementary file 5 — Reporting Summary [file 41467_2020_19102_MOESM5_ESM.pdf]

nature research

Corresponding author(s): Yan Zhu

Last updated by author(s): Jul 11, 2020

Reporting Summary

Nature Research wishes to improve the reproducibility of the work that we publish. This form provides structure for consistency and transparency in reporting. For further information on Nature Research policies, see Authors & Referees and the Editorial Policy Checklist .

Statistics

For all statistical analyses, confirm that the following items are present in the figure legend, table legend, main text, or Methods section.

n/a	Confirmed	
☐	☒	The exact sample size (*n*) for each experimental group/condition, given as a discrete number and unit of measurement
☒	☐	A statement on whether measurements were taken from distinct samples or whether the same sample was measured repeatedly
☐	☒	The statistical test(s) used AND whether they are one- or two-sided<br>*Only common tests should be described solely by name; describe more complex techniques in the Methods section.*
☒	☐	A description of all covariates tested
☒	☐	A description of any assumptions or corrections, such as tests of normality and adjustment for multiple comparisons
☐	☒	A full description of the statistical parameters including central tendency (e.g. means) or other basic estimates (e.g. regression coefficient) AND variation (e.g. standard deviation) or associated estimates of uncertainty (e.g. confidence intervals)
☐	☒	For null hypothesis testing, the test statistic (e.g. *F*, *t*, *r*) with confidence intervals, effect sizes, degrees of freedom and *P* value noted<br>*Give P values as exact values whenever suitable.*
☒	☐	For Bayesian analysis, information on the choice of priors and Markov chain Monte Carlo settings
☒	☐	For hierarchical and complex designs, identification of the appropriate level for tests and full reporting of outcomes
☒	☐	Estimates of effect sizes (e.g. Cohen's *d*, Pearson's *r*), indicating how they were calculated

Our web collection on statistics for biologists contains articles on many of the points above.

Software and code

Policy information about availability of computer code

Data collection	1. Behavioral data were collected using a custom script in MATLAB 2018a (MathWorks), which gather video frames at a set frame rate from web cameras. 2. Imaging data were obtained on a Leica TCS SP8 confocal microscope.
Data analysis	Extracting flies and analyzing their distribution in each video frame were accomplished by a custom script in MATLAB 2018a (MathWorks).<br>Statistical analyses were conducted using Prism 6 (GraphPad software Inc).<br>Microscopy image data for quantification of fluorescent signal were loaded using the Bio-Formats 6.5.0 (Bio-Formats library).<br>3D image construction was processed in Fiji using 3D Viewer plugin.

For manuscripts utilizing custom algorithms or software that are central to the research but not yet described in published literature, software must be made available to editors/reviewers. We strongly encourage code deposition in a community repository (e.g. GitHub). See the Nature Research guidelines for submitting code & software for further information.

Data

Policy information about availability of data

All manuscripts must include a data availability statement. This statement should provide the following information, where applicable:

- Accession codes, unique identifiers, or web links for publicly available datasets
- A list of figures that have associated raw data
- A description of any restrictions on data availability

Figures 1C–E, 2A-E, 3B-H, 3J, 4B-D, 5B, 5D, 5G, 6A, 6F, 6G, 6I and Supplementary Figures S1B-E, S1G-H, S2B-D, S3, S4B-C, S5, S6, S7, S8, S11, S12, S13D, S14, S15B, S15D, and S19C are provided as a Source Data file with this paper. Additional raw data that support the findings of this study are available upon request.

Field-specific reporting

Please select the one below that is the best fit for your research. If you are not sure, read the appropriate sections before making your selection.

☒ Life sciences ☐ Behavioural & social sciences ☐ Ecological, evolutionary & environmental sciences

For a reference copy of the document with all sections, see nature.com/documents/nr-reporting-summary-flat.pdf

Life sciences study design

All studies must disclose on these points even when the disclosure is negative.

Sample size	The sample size was always equal to or more than 5 for imaging experiment and 7 for social approach analysis, denatonium conditioning experiment, and male-male courtship conditioning.
Data exclusions	No data were excluded.
Replication	All experiments were repeated at least 3 times on different days with different batch of flies. The variation was minimized by optimizing the test condition with pilot studies in the early phase of this study.
Randomization	Flies of the same genotype were sorted and collected into a common vial, from which they were randomly selected for all experiments.
Blinding	Distribution of the flies were video-recorded and the files were numbered. The operators were blind to the test groups or conditions. We used computer code for quantification.

Reporting for specific materials, systems and methods

We require information from authors about some types of materials, experimental systems and methods used in many studies. Here, indicate whether each material, system or method listed is relevant to your study. If you are not sure if a list item applies to your research, read the appropriate section before selecting a response.

Materials & experimental systems

n/a	Involved in the study	
☐	☒	Antibodies
☒	☐	Eukaryotic cell lines
☒	☐	Palaeontology
☐	☒	Animals and other organisms
☒	☐	Human research participants
☒	☐	Clinical data

Methods

n/a	Involved in the study	
☒	☐	ChIP-seq
☒	☐	Flow cytometry
☒	☐	MRI-based neuroimaging

Antibodies

Antibodies used	1. anti-nc82 (DSHB, CAT# AB_2314866)<br>2. Rhodamine (TRITC) Conjugated Goat anti-Mouse IgG (H+L) (ZSGB-BIO, CAT# ZF-0313)
Validation	1. anti-nac82, mouse Bruchpilot Brp antibody, Developmental Studies Hybridoma Bank, https://dshb.biology.uiowa.edu/nc82<br>2. Rhodamine (TRITC) Conjugated Goat anti-Mouse IgG (H+L) , ZSGB-Bio Co., Ltd., http://www.zsbio.com/product/ZF-0313

Animals and other organisms

Policy information about studies involving animals; ARRIVE guidelines recommended for reporting animal research

Laboratory animals	Drosophila melanogaster, Drosophila simulans, Drosophila sechellia, Drosophila yakuba, and Drosophila repleta, wild type laboratory strains and transgenic strains, genotype, age and sex specified in detail in the methods.
Wild animals	This study did not involve wild animals.
Field-collected samples	This study did not involve samples collected from the field.
Ethics oversight	As a general practice, no ethical approval or guidance was required for the insects included in this study.

Note that full information on the approval of the study protocol must also be provided in the manuscript.
